# Histological and Immunohistochemical Analyses of Repair of the Disc in the Rabbit Temporomandibular Joint Using a Collagen Template

**DOI:** 10.3390/ma10080924

**Published:** 2017-08-09

**Authors:** Kuo-Hwa Wang, Wing P. Chan, Li-Hsuan Chiu, Yu-Hui Tsai, Chia-Lang Fang, Charn-Bing Yang, Kuan-Chou Chen, Hung-Li Tsai, Wen-Fu Lai

**Affiliations:** 1 Graduate Institute of Clinical Medicine, College of Medicine, Taipei Medical University, Taipei 11031, Taiwan; khwang117@gmail.com (K.-H.W.); dryangcb@gmail.com (C.-B.Y.); kuanchou@tmu.edu.tw (K.-C.C.); 2 Department of Radiology, School of Medicine, College of Medicine, Taipei Medical Univesity, Taipei 110, Taiwan; wp.chan@msa.hinet.net; 3 McLean Imaging Center, Harvard Medical School, Belmont, MA 02478, USA; lchiu@mclean.harvard.edu; 4 Graduate Institute of Medical Sciences, Taipei Medical University, Taipei 11031, Taiwan; cmbyht18@tmu.edu.tw (Y.-H.T.); d102094012@tmu.edu.tw (H.-L.T.); 5 Department of Pathology, School of Medicine, College of Medicine, Taipei Medical University, Taipei 11031, Taiwan; ccllfang@tmu.edu.tw; 6 School of Dentistry, College of Dentistry, Taipei Medical University, Taipei 11031, Taiwan

**Keywords:** temporomandibular joint disc, reconstituted collagen template, tissue regeneration

## Abstract

A previous study demonstrated that the reconstituted type I collagen matrix extracted from rabbit tendons enabled the TMJ disc to regenerate in the rabbit. The aim of this study was to investigate changes in the extracellular matrix (ECM) and mechanisms of regeneration in the TMJ disc. In 36 New Zealand rabbits that underwent a partial discectomy, discs were replaced with reconstituted collagen templates for 3 months. A histological analysis showed that moderate to severe degeneration appeared in partially discectomized joints without implantation. In contrast, discs experienced regeneration of reconstituted collagen template implantation and the joint returned to normal function. Cells in the regenerative tissue expressed ECM, and fibers became regular and compact due to tissue remodeling over time. Reparative cells differentiated into chondroblasts, and showed highly dense pericellular fibers. The morphology and collagen composition of the disc and condyle in the 3-month experimental group were similar to those of normal tissues. In conclusion, the reconstituted collagen template facilitated the regeneration of surgically discectomized discs. Type I and type II collagens play a crucial role in the regeneration of articular discs.

## 1. Introduction

Temporomandibular joint (TMJ) disorders (TMDs) are major causes of degenerative changes in the disc and condylar cartilage. If pathologic changes persist following conservative or non-surgical treatment [1], degeneration or perforation of the disc might occur, and result in surgical removal of the disc [2,3]. Surgical techniques such as a discectomy, arthroplasty, and condylectomy have been used to provide migration of blood cells and mesenchymal stem cells from the bone marrow to the defect [4]. These surgical treatments achieve fibrocartilagenous healing that may improve symptoms; however, the reparative tissues often lead to limitations of joint function [5].

Previously, we designed a reconstituted collagen template for cartilage regeneration of the rabbit TMJ disc after a partial discectomy [6,7], and established a model of in vitro proliferation of chondrocytes in a collagen matrix [8,9]. Hematoxylin and eosin (HE) staining results showed that the template induced regeneration of the articular disc. However, the mechanism of disc regeneration is still not well understood. Also, given the scarcity of studies morphologically focusing on the types of collagen in TMJ disc regeneration, a better understanding of the structural mechanisms between collagens and disc regeneration of the TMJ is greatly needed.

The purpose of this study was to further determine the collagen matrix pattern of the regenerative disc and joint cartilage in experimental discectomies of the TMJ in rabbits. Immunohistochemical evaluations of discectomized discs, repaired with a reconstituted collagen template, were employed. Results showed a similar collagen distribution to that of normal joint tissue.

## 2. Materials and Methods

### 2.1. Rabbits

Thirty-eight adult New Zealand male rabbits were housed in well-ventilated cages and fed a regular diet. The average age of the rabbits was 3 months, and their average weight was 2.0 kg. All animal husbandry and handling procedures including animal monitoring, diet, primary enclosures, and environmental control, followed standard operating procedures in accordance with the *Animal (Scientific Procedures) Act* 1986.

### 2.2. Reconstituted Collagen Templates

Reconstituted collagen templates were prepared as previously described [6,7]. Type I and type II collagens were extracted and purified from tendons and cartilage of New Zealand white rabbits, as previously described in our laboratory [10].

### 2.3. Experimental Design

Thirty-eight animals were divided into experimental (collagen template implantation) (*n* = 18), untreated (without implantation) (*n* = 18), and sham-operated groups (*n* = 2). The experimental and untreated groups comprised 18 animals in each group: 6 animals under observation for 3 months, 6 animals for 2 months, 6 animals for 1 month, respectively, after the partial discectomy. The remaining 2 animals comprised the sham-operated group. The unoperated sides served as intact controls.

### 2.4. Surgical Techniques

Thirty-six adult New Zealand male rabbits underwent a partial posterolateral discectomy of the TMJ disc. Two sham-operated rabbits were only opened up and closed without specific removal of joint tissue. The TMJ region of each rabbit was shaved and prepped with povidone-iodine solution under general anesthesia with ketamine (35 mg/kg) and Citosol (50 mg/kg), followed by lidocaine infiltration. Half of the operations were performed on the right side and the other half on the left side to avoid the operation side as a confounder in the biostatistics.

A curvilinear incision was made along the zygomatic arch extending from the lateral aspect of the canthus to just anterior to the external auditory meatus [7]. The overlying tissue was flapped inferiorly, and the TMJ was exposed. Following the incision along the articular fossa and the eminence, a 0.5-cm segment of the zygomatic process was removed. The capsule tissue was reflected, and the disc was identified.

A partial discectomy (3.5 × 6.0 mm^2^) was performed on the posterolateral portion of the TMJ disc. The reconstituted collagen templates or dermal grafts were immediately implanted as the disc-replacement and fixed, after which the articular capsule was closed with 4-0 silk non-resorbable sutures. The skin incision was then closed with 4-0 silk non-resorbable sutures.

After surgery, the body weight of each rabbit was measured weekly to determine whether clinical problems in the TMJ were reflected by food intake. From 1 to 3 months after surgery, animals were sacrificed by a lethal intraperitoneal pentobarbital injection (60 mg/kg); the TMJ tissue with implants was excised en bloc and processed for gross, histological, and immunohistochemical (IHC) evaluations.

### 2.5. Histology Preparation

TMJ tissues with implants were coronally en bloc excised. Specimens were fixed in formalin, and decalcified with DECAL-RAPID (National Diagnostics, Atlanta, GA, USA) for 10 h. Tissues were then embedded in paraffin and serially sectioned (Sakura Sledge microtome, Sakura Finetek Japan, Tokyo, Japan) at 5~10 μm. Tissue sections were stained with HE. The tissue regeneration and/or fibrosis of the defective area were evaluated histologically including host response, tissue response to the surgical trauma, and tissue regeneration.

### 2.6. Immunohistochemistry and Relative Quantification

Serial sections of each sample were incubated with the primary antibody (anti-collagen type I and anti-collagen type II, ThermoFisher Sientific, Waltham, MA, USA) or control blank serum. The antigen–antibody was further incubated with a horseradish peroxidase secondary antibody. Complexes were revealed with diaminobenzidine (DAB) to determine the collagen typing changes. The expression levels of type I, and II collagens were evaluated using relative immunochemical staining TMJ disc tissue sections, which compare between different samples based on objective data [11]. Images were acquired sequentially and analyzed by Aperio Scanscope Console software (Informer Technologies, Inc., Shingle Springs, CA, USA). TIFF images were processed using Photoshop 4.0 software (Adobe, San Jose, CA, USA).

### 2.7. Statistical Analysis

The immunostaining intensities of type I, and II collagens were analyzed using at least three samples and counted for each of the two groups. Statistical computations were performed using Student *t* test. Data are reported as the mean ± S.D. *p* < 0.05 was considered statistically significant.

## 3. Results

### 3.1. Sham-Operated Group

#### 3.1.1. Histological Evaluation

The normal articular disc was coronally concave in shape and composed of dense connective tissue. Synovial fibroblasts and chondroblasts were found in the dense connective tissue.

**Figure 1:**
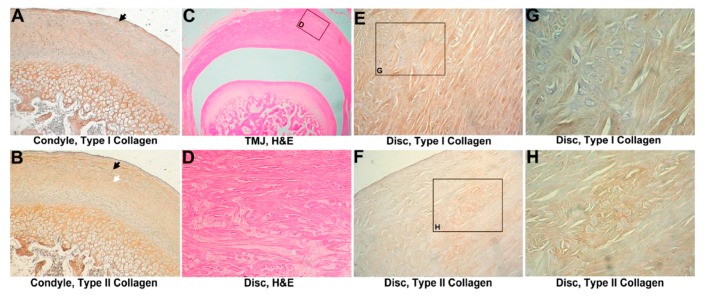
Analysis of the intact control. (**A**) Immunohistochemistry of type I collagen on normal temporomandibular joint (TMJ) cartilage, 800×. Type I collagen was expressed on the cartilage surface, bone, and proliferation zone (black arrow head); (**B**) Immunohistochemistry of type II collagen on normal TMJ cartilage, 800×. Type II collagen was expressed at the hyaline zone (white arrowhead) and proliferation zone (black arrow head); (**C**) hematoxylin and eosin (HE) analysis of a normal TMJ, 160×. Normal architecture of the disc, condyle, and cartilage; (**D**) HE analysis of the normal TMJ disc, 1600×. Chondrocytes, surrounded by wavy fibers, are scattered in the disc; (**E**) Immunohistochemistry of type I collagen in the normal TMJ disc, 1600×. Type I collagen was noted in wavy fibers; (**F**) Immunohistochemistry of type II collagen on a normal TMJ disc, 1600×. Type II collagen was expressed around chondrocyte-like cells; (**G**) Immunohistochemistry of type I collagen in the normal TMJ disc, 3200×. Magnification of (**E**); (**H**) Immunohistochemistry of type II collagen in the normal TMJ disc, 3200×. Magnification of (**F**).

The condyle was composed of cancellous bone covered by a thin layer of compact bone. The outer surface on the condyle was covered with fibrocartilage tissue (Figure 1C). Between the fibrocartilage layer and compact bone was a layer of hyaline cartilage (Figure 1C). The fibrocartilage layer consisted of a layer of fibrous tissue with scattered chondroblasts (Figure 1C,D). No significant difference between the unoperated and sham-operated groups was found. The TMJ disc was composed of wavy and thick collagen fibers where disc chondroblasts were growing (Figure 1D).

#### 3.1.2. IHC Evaluation

In normal TMJ cartilage, type I collagen was expressed on all fibrocartilage layers, and type II collagen was mainly expressed in the hypertrophic zone (Figure 1A,B). In the disc, type I collagen was stained within wavy fiber structures (Figure 1E,G), indicating that type I collagen was expressed in the form of woven collagen bundles within the tissue. Type II collagen was expressed around clusters of polygonal chondroblasts in the outer part of the disc (Figure 1F,H). The relative intensities of type I and type II collagen expressions in condyle were measured. Approximately, the ratio of the collagen immunostaining intensity of type II to type I is 3.7 to 1 (Figure S1).

### 3.2. Untreated Group

#### 3.2.1. Histological Evaluation

**Figure 2:**
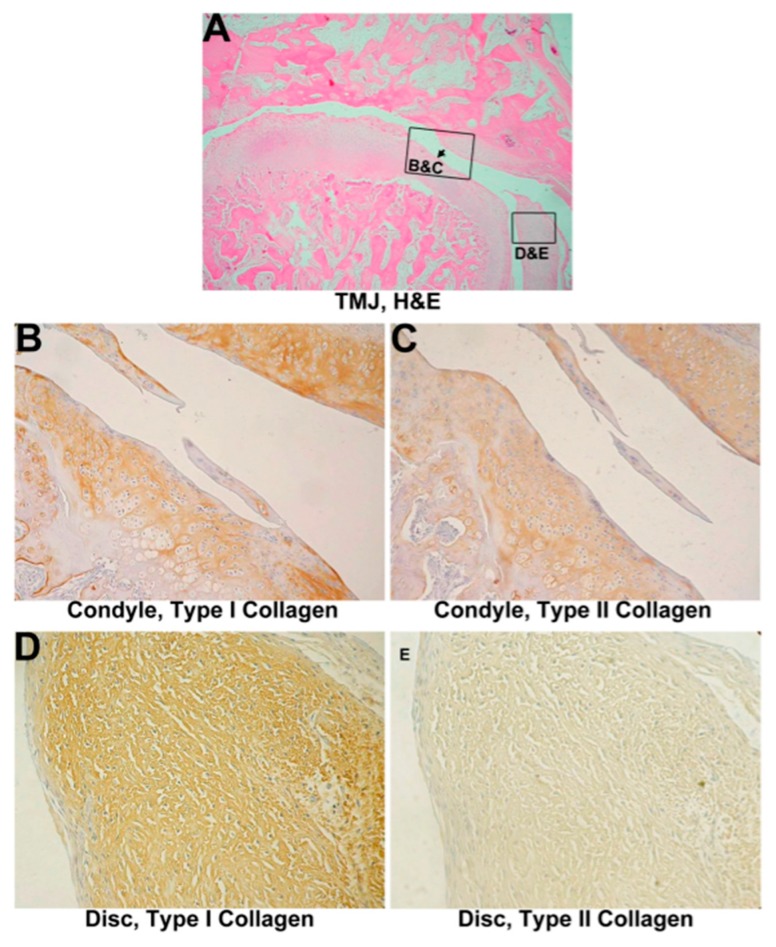
Histological analysis of a 1-month discectomied TMJ without implantation. (**A**) One-month group, 320×. Mild chondroblast proliferation in the cartilage was evident. The condyle surface appears erosive and moderately deformed (black arrowhead). The remainder of the disc shows fibrous degeneration; (**B**) Immunohistochemistry of type I collagen on 1-month untreated TMJ cartilage, 800×. Type I collagen expression increased and surrounded proliferating cells on the cartilage surface; (**C**) Immunohistochemistry of type II collagen on 1-month untreated TMJ cartilage, 800×. Type II collagen, like type I, surrounded proliferating cells; (**D**) Immunohistochemistry of type I collagen on a 1-month untreated TMJ disc, 1600×. Type I collagen showed nearly normal expression; (**E**) Immunohistochemistry of type II collagen on a 1-month untreated TMJ disc, 1600×. Minimal expression was observed.

All 18 untreated discectomized specimens were evaluated after 1 month, 2 months, and 3 months. Six discectomied joints in the 1-month group showed mild to moderate fibrosis on the condyle and the remainder of the disc at 1 month. Cartilage erosion and enlargement were found on the outer layer of the condyle and tympanic fossa. Degeneration was noted in the disc. Chondroblast proliferation and cluster formation were predominantly found in the outer layer of cartilage in both the condyle and tympanic fossa (Figure 2A).

**Figure 3:**
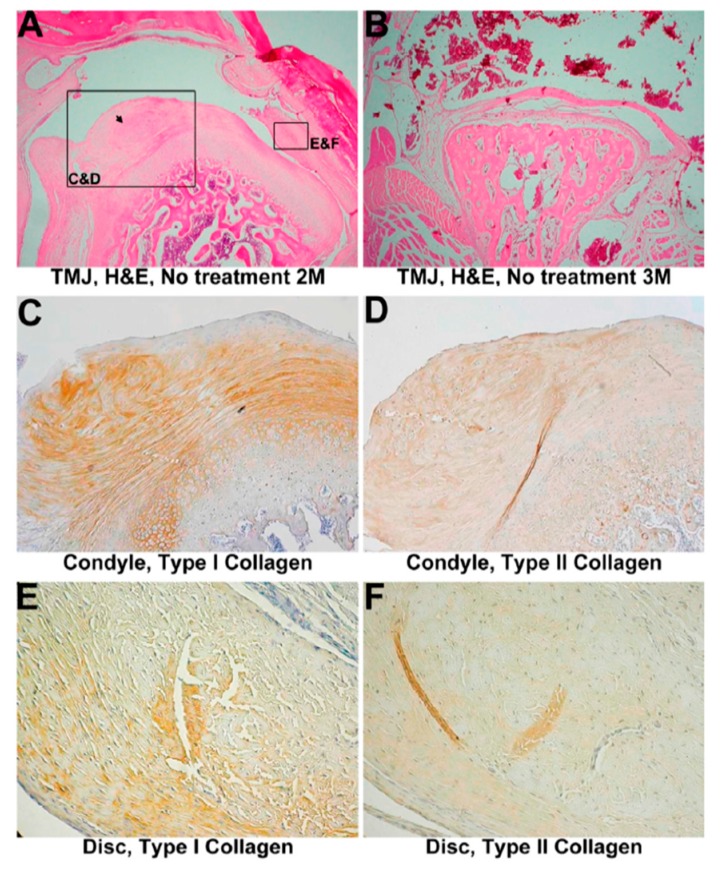
Histological analysis of the discectomied TMJ without implantation. (**A**) Two-month group, 320×. The remainder of the disc is characterized by fibrillary degeneration, and it was covered by a synovial lining. Marked cartilage enlargement and fragmentation, and fibrous degeneration of both the condyle (black arrowhead) and tympanic fossa was found; (**B**) Three-month group, 320×. The condyle shows complete erosion of cartilage from the subchondral bone; (**C**) Immunohistochemistry of type I collagen in 2-month untreated TMJ cartilage, 800×. Type I collagen was greatly expressed in enlarged cartilage; (**D**) Immunohistochemistry of type II collagen in 2-month untreated TMJ cartilage, 800×. The expression of type II collagen had significantly decreased in enlarged cartilage; (**E**) Immunohistochemistry of type I collagen in a 2-month untreated TMJ disc, 1600×. Like the normal disc, type I collagen expression was found in fibers; (**F**) Immunohistochemistry of type II collagen in the 2-month untreated TMJ disc, 1600×. Minimal type II collagen expression surrounds cells.

In the 2 month-group, the condyle and tympanic fossa showed moderate erosion, which was accompanied by marked cartilage enlargement and fragmentation in the whole group of six animals. Chondroblast proliferation was scattered in the cartilage (Figure 3A) without cluster formation. Significant fibrous tissue formation was noticed at the condyle and tympanic fossa with unregular fiber alignment. The condyle cartilage showed moderate to severe enlargement and fragmentation, while the remaining disk showed fibrillary degeneration and was covered by a synovial lining.

Cartilage was completely eroded from the subchondral bone in the 3-month group. The remainder of the disc was torn out, and the articular surface was covered with fibrous connective tissue in five of six joint specimens (Figure 3B). The sixth joint exhibited severe cartilage erosion of the condyle. All six joints exhibited severe erosion of the tympanic fossa.

#### 3.2.2. IHC Evaluation

Type I collagen showed an increase on the cartilage surface and in the zone of chondroblast proliferation. The layer of hyaline cartilage expressed more type I collagen than that expressed in normal specimens after 1 month or 2 months after a discectomy (Figure 2B or Figure 3C). A decrease in type II collagen around proliferating chondroblasts was also found in the cartilage area of the mandibular condyle process and tympanic fossa in the arthritic group (Figure 2C or Figure 3D). A different appearance was found in the remainder of the disc compared to the condyle. Type I collagen did not change (Figure 2D or Figure 3E); however, type II collagen showed only minimal change (Figure 2E or Figure 3F). The partial discectomized joint without implantation exhibited obviously inflammation after 2 months. Type I collagen remarkably increased in both the enlarged and fragmented portions of the condyle, whereas type II collagen showed a mild increase in the condyle and was almost not found in the disc. The relative quantification of collagen expression showed, in parallel with the immunostaining in the condyle, a minor increase of type I collagen expression at 1 month, followed by a significant increase of type I collagen at 2 months. Gradually decreased expressions of type II collagen were examined in 1-month and 2-month groups (Figure S1).

### 3.3. One-Month Group

#### 3.3.1. Histological Evaluation

**Figure 4:**
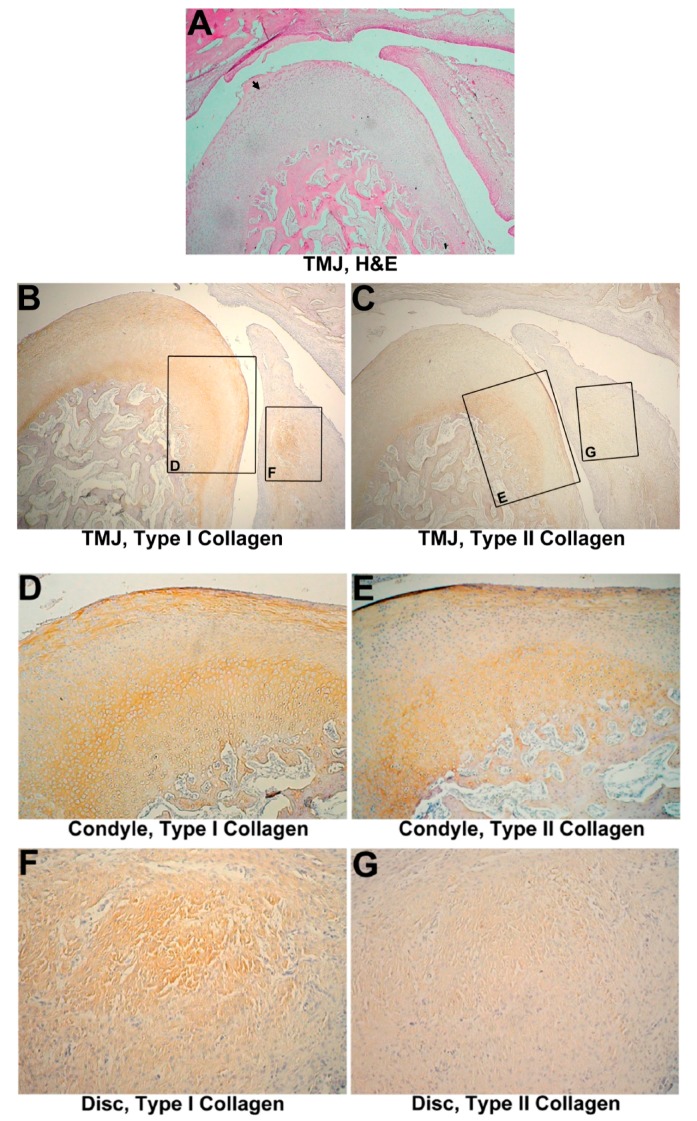
Discectomied TMJ with implantation in 1 month. (**A**) HE analysis of the temporomandibular joint (TMJ) with a 1-month reconstituted collagen template, 320×. Cartilage exhibited mild chondroblast proliferation with minimal erosion (black arrow). The disc shows fibrous degeneration; (**B**) Immunohistochemistry of type I collagen in a 1-month treated TMJ, 320×; (**C**) Immunohistochemistry of type II collagen in a 1-month treated condyle; 320× (**D**) Immunohistochemistry of type I collagen in 1-month treated TMJ cartilage, 800×. Type I collagen was increasingly expressed on the cartilage surface; (**E**) Immunohistochemistry of type II collagen in 1-month treated TMJ cartilage, 800×. Mild type II collagen expression surrounds proliferating cells on the surface; (**F**) Immunohistochemistry of type I collagen in a 1-month treated TMJ disc, 1600×. Type I collagen showed increasing expression; (**G**) Immunohistochemistry of type II collagen in a 1-month treated TMJ disc, 1600×. Decreased expression was observed.

A mild lateral regeneration from the medial side towards lateral side that covered one-third of the condyle in the implant group is shown in Figure 4A. The regenerated part appeared thinner and more primitive compared to the initial part. Chondroblast proliferation and clustering were noticed. No disc ossification or calcification was observed in discs of either the experimental or control groups. The condyles of the experimental group showed mild erosion, whereas the control group showed mild to moderate erosion. No cartilage fragmentation was observed in the condyles of the experimental group and control group.

The remainder of the reconstituted collagen template was noted at the surgical site. Lymphohistiocytic infiltration and foreign body giant cells were also present.

#### 3.3.2. IHC Evaluation

Mild type I collagen expression was found on both the condyle and disc (Figure 4B,D). Type II collagen decreased in the cartilage or disc (Figure 4C,E,G) compared to the intact control. The relative staining intensities of type I and type II collagen expressions at the condyle reflect the staining outcomes. The expression levels demonstrated an increase in type I collagen and a decrease in type II collagen (Figure S1). A similar type I collagen/type II collagen pattern was noted compared to the 1-month group without collagen template implantation.

### 3.4. Two-Month Group

#### 3.4.1. Histological Evaluation

**Figure 5:**
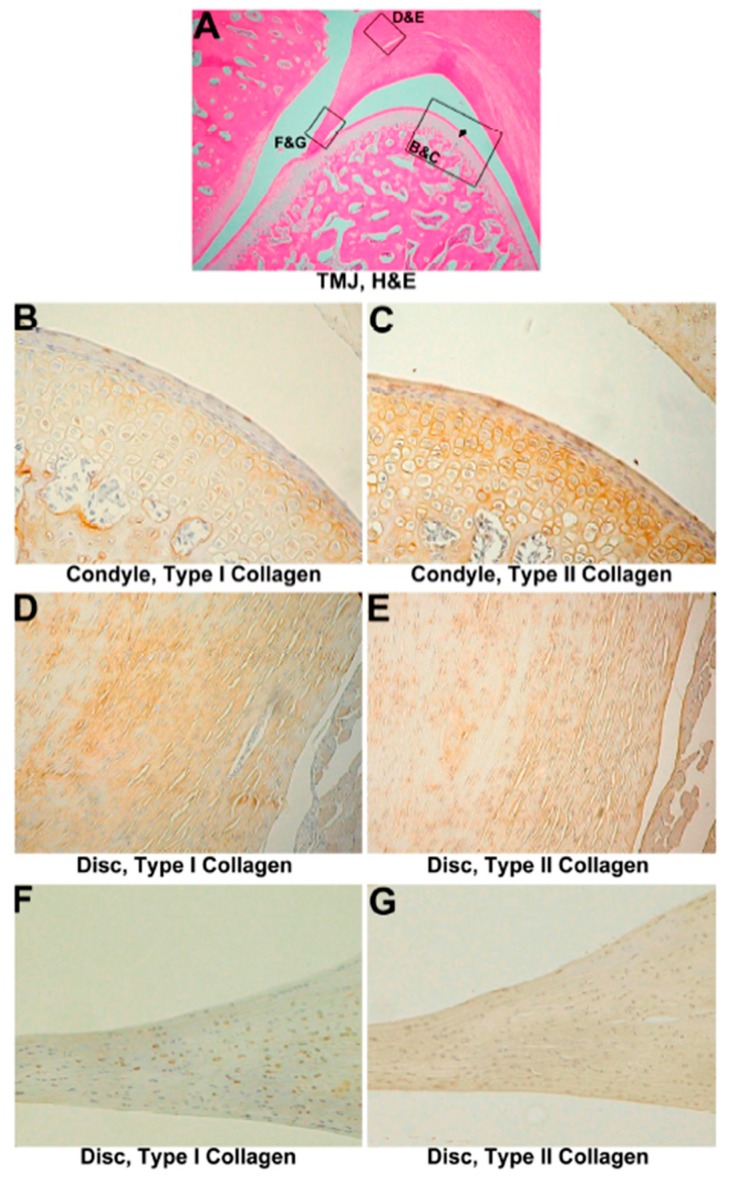
Discectomied TMJ with implantation after 2 months. (**A**) HE stain, 320×. The disc appears to be somewhat thicker than that of the 1-month group. No erosion is present on either the tympanic fossa or condyle (black arrowhead); (**B**) Immunohistochemistry of type I collagen on 2-month treated TMJ cartilage, 800×. Type I collagen showed a slightly increasing expression; (**C**) Immunohistochemistry of type II collagen in 2-month treated TMJ cartilage, 800×. Expression of type II collagen was decreased compared to the intact control; (**D**) Immunohistochemistry of type I collagen in a 2-month treated TMJ disc, 1600×. Type I collagen was expressed on wavy fibers; (**E**) Immunohistochemistry of type II collagen in a 2-month treated TMJ disc, 1600×. Type II collagen was expressed surrounding chondrocyte-like cells; (**F**) Immunohistochemistry of type I collagen in a 2-month treated TMJ disc tip, 1600×. Type I collagen was expressed in chondrocyte-like cells; (**G**) Immunohistochemistry of type II collagen in a 2-month treated TMJ disc tip, 1600×. Less type II collagen was observed.

Disc regeneration covering more than one-third of the condyle was noted. The regenerated disc of the 2-month group appeared a little thicker than that of the 1-month group (Figure 5A). Significant chondroblast proliferation and clustering were observed at the condyle. Regular fiber alignment and a cartilage transitional layer were noticed at the condyle similar to the intact control. No fibrosis, ossification, or calcification was found on the disc (Figure 5A). No fibrous layer erosion was observed on the condyle or the tympanic fossa of the temporal bone.

#### 3.4.2. IHC Evaluation

In the 2-month group, the remainder of the disc expressed type I collagen fibers in a wavy appearance (Figure 5D), and expressed type II collagen around chondroblasts (Figure 5E,G). Type I collagen was also expressed in cells at the cutting surface of the disc (Figure 5F). The type I/type II collagen pattern was more similar to that of normal specimens in both the joint cartilage and disc (Figure 5B,C), indicating that the template and regenerated disc prevented tearing of the cartilage and inflammation of the joint. The relative staining intensities of type I and type II collagen expressions at the condyle were measured. An increase in type I collagen and a mild decrease in type II collagen compared to the intact control were still noted (Figure S1). However, a relative lower type I collagen expression and a higher type II collagen expression level were noted compared to the 2-month group without collagen template implantation. These results indicate that the template had a protective capacity, diminished tearing of the joint and promoted regeneration of the cartilage.

### 3.5. Three-Month Group

#### 3.5.1. Histological Evaluation

**Figure 6:**
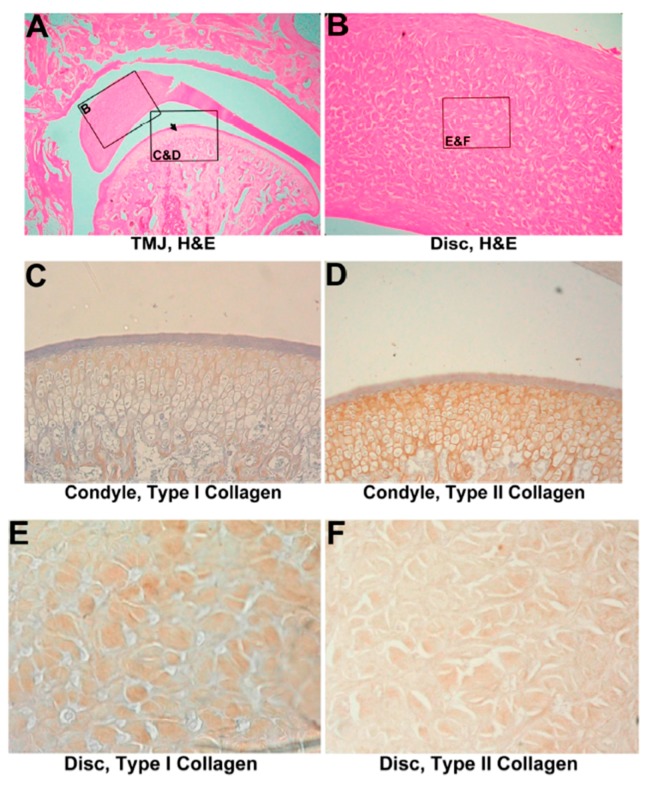
Discectomied TMJ with implantation in 3 months. (**A**) HE analysis of the temporomandibular joint (TMJ) with a 3-month reconstituted collagen template, 320×. New collagen bundles appeared in the disc and had adhered to the tympanic fossa; the condyle appears normal with a smooth condylar surface (black arrowhead) (**B**) HE analysis of the TMJ disc with a 3-month reconstituted collagen template, 1600×. The direction and arrangement of new fibers differed from those of old fibers; (**C**) Immunohistochemistry of type I collagen in 3-month treated TMJ cartilage, 1600×. Type I collagen had recovered to a normal state; (**D**) Immunohistochemistry of type II collagen in 3-month treated TMJ cartilage, 1600×. Expression of type II collagen was normal; (**E**) Immunohistochemistry of type I collagen in a 3-month treated TMJ disc, 3200×. Type I collagen was expressed in fibers; (**F**) Immunohistochemistry of type II collagen in a 3-month treated TMJ disc, 3200×. Type II collagen was expressed randomly in fibers adjacent to cells.

Discs of the experimental group showed apparent regeneration 3 months after implantation (Figure 6A). At the condyle, significant chondroblast proliferation and clustering were observed. Mature hyaline cartilage layers including the tangential zone, transitional zone, and radial zone were observed, which is similar to the intact control. New collagen bundles had formed and adhered to the tympanic fossa. Primitive chondrocyte-like cells were scattered in mature collagen bundles at the disc, when new collagen bundles appeared slightly disoriented compared to the intact control (Figure 6B).

#### 3.5.2. IHC Evaluation

After 3 months of implantation, the regenerated disc had almost covered the TMJ cartilage with a normal collagen pattern and layers (Figure 6C,D). Type I collagen of the regenerated disc was revealed on collagen bundles (Figure 6E), and type II collagen was expressed around fiber sections and chondrocyte-like cells (Figure 6F). The relative staining intensities of type I and type II collagens at the condyle were measured. Type I collagen and type II collagen levels were similar to that of the intact control, while a significant increase of type II expression level was examined compared to the 1-month and 2-month implantation groups. The ratio of type I to II collagen staining intensity is approximately 3.4 to 1, which is close to the intact control (Figure S1). These indicate that the template showed a regeneration capacity and some newborn cells had repaired the injured tissue by synthesizing type I and type II collagens. It is noteworthy that type I collagen immunostaining intensity decreases and type II increases, during the 1-month, 2-month, and 3-month implantation period. The collagen pattern appeared nearly the same as normal tissues.

## 4. Discussion

Dermal grafts can relieve the pain of TMDs and provide protection to maintain cartilage integrity [12,13], although they are insufficient to induce the regeneration of the disc [14]. Since 1989, temporalis muscle flaps have been the usual treatment for TMDs [15,16], somewhat like derma grafts, which provide no disc repair. In our study, the reconstituted collagen template formed optimal 50~150-μm pores that were suited for cell ingrowth [17]. Attempts have been made to apply stem cells [18,19], cytokines [20,21,22], platelet-rich plasma [23] for TMJ regeneration, however, no predictable therapy is currently available in the clinic, and thus our implants provide a method to regenerate TMJ discs. Our previous study has proposed that the reconstituted collagen template may exhibit a cushioning effect to minimize physical damage to the joint, and acts as a provisional extracellular matrix to facilitate proliferation and differentiation of disc cells and contribute to regeneration of the disc [7]. In this current study, we further demonstrated that the regenerated TMJ disc not only exhibited a normally histological morphology, but also expressed collagen compositions similar to those of normal tissues in the 3-month experimental group. These outcomes indicated that the collagen template not only provided mechanical resistance to protect the cartilage, but also provided cartilaginous microenvironment to recruit reparative cell ingrowth. These effects facilitate the regeneration potency better than previous traditional implants.

Immune responses cause major problems for exogenous implant applications [24]. Our previous study reported that a reconstituted collagen template lasted 3 months in vivo [7]. Lymphohistocytic infiltration and foreign body giant cells were found within the collagen template in the first 2 months. This immune response ceased with complete degradation of the template in the third month. In the present study, the inflammatory response induced by the exogenous template did not cause obvious cartilage destruction, although inflammation was noted in the beginning of TMJ osteoarthritis. The reconstituted collagen template is not only required to remain long enough until complete disc regeneration, but it also moderates exogenous material-induced inflammation.

The amount of collagen loss and synthesis plays a crucial role in the osteoarthritic change or repair. Our data showed that after 3 months of implantation, the regenerated cartilage showed normal collagen-abundant layers similar to the intact control (Figure 6C,D). The regenerated disc showed type I collagen bundles (Figure 6E), with type II collagen expressed around chondrocyte-like cells (Figure 6F). The immunostaining did not distinguish the implanted reconstituted-collagen from the newly formed collagen matrix. However, the IHC evaluation showed that type I collagen immunostaining intensity decreases and that of type II collagen increases between experimental groups from 1 month to 3 months. The ratio of type I to II collagen immunostaining is 3.4 to 1 in the 3-month group, which is close to the intact control (Figure S1). The data demonstrates that with template implantation, the regenerated TMJ disc in the cartilaginous microenvironment facilitates cells to express chondrogenic features and built-up type II collagen-abundant matrices. In contrast, without implantation, the collagen-abundant matrix may gradually decrease after 1 to 2 months, and the TMJ was totally destroyed after 3 months. These outcomes imply that type I and type II collagen content play a crucial role in the regeneration of articular discs.

Recently, Brown et al. used the porcine urinary bladder matrix (UBM) as a disc material. Results showed TMJ protection and disc regeneration [25,26]. UBM is mainly comprised of type I collagen and has similar components as the reconstituted collagen template [27,28]. In the canine TMJ, UBM protected TMJ cartilage and triggered cell and vessel ingrowth to form new disc tissues after 6 months of treatment [25,26]. The UBM and reconstituted collagen template demonstrate that acellular ECM-based matrices facilitate disc regeneration in the TMJ. However, UBM, like the reconstituted collagen template, also induced a foreign body reaction [25,26]. Long-term inflammation needs to be moderated and controlled, although the immune response decreased with time.

Autogenous grafts, such as temporalis flaps [15,29], auricular cartilage [30,31], and dermis-fat grafts [13,32], have been used in animals with a discectomy and have shown varied success rates. However, they require more surgery and have no inductive effect on disc regeneration. Alloplastic materials, such as sialastic [33] and Teflon [34] implants, have been withdrawn because of severe foreign body reactions and bone erosion [33,35]. Recently, exogenous acellular ECM-based materials [36], such as reconstituted collagen templates and UBM, have shown more predictable regenerative capacities compared to other implants. If immune activation by reconstituted collagen templates can be resolved and biodegradation can be controlled until the completion of disc regeneration, acellular ECM-based materials will be potential candidates for TMD therapy.

In summary, the reconstituted collagen template protected TMJ cartilage from trauma and induced disc regeneration 3 months after a discectomy. With reconstituted collagen templates, TMJ cartilage maintained normal collagen expression, and recruited disc cells and chondrocyte-like cells that expressed type I and type II collagens respectively to regrow the excised disc. The reconstituted collagen template can be a potential candidate for implantation for TMD therapy, although immune activation needs to be controlled. Further studies will focus on controlling the biodegradation rate and alleviating immune responses.

## Supplementary Materials

The following are available online at www.mdpi.com/1996-1944/10/8/924/s1.
