# Supplementary material for: Histological and Immunohistochemical Analyses of Repair of the Disc in the Rabbit Temporomandibular Joint Using a Collagen Template
Source: Materials (Basel). 2017 Aug 9;10(8):924. doi: 10.3390/ma10080924 (PMC5578290; doi:10.3390/ma10080924)
Supplement: Supplementary file 1 [file materials-10-00924-s001.pdf]

Supplementary Materials: Histological and Immunohistochemical Analyses of Repair of the Disc in the Rabbit Temporomandibular Joint Using a Collagen Template

Kuo-Hwa Wang [1,†], Wing P. Chan [2,†], Li-Hsuan Chiu [3], Yu-Hui Tsai [4], Chia-Lang Fang [5], Charn-Bing Yang [1], Kuan-Chou Chen [1], Hung-Li Tsai [4,‡] and Wen-Fu Lai [1,3,6,*]

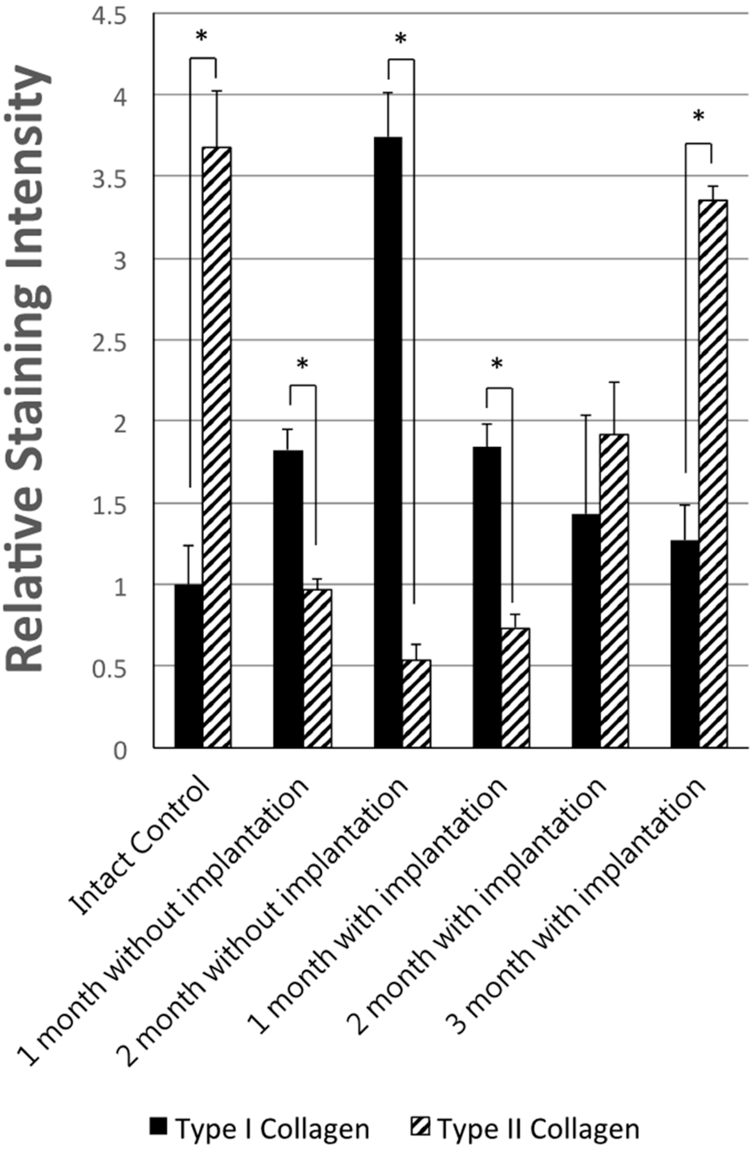

Figure S1. Relative immunostaining intensities of type I and II collagen in the condyle with and without collagen template implantation.

The ratio of type II to type I collagen-staining level was measured approximately 3.7 to 1 in the intact control. Type I collagen gradually increased at 1-month and 2-month after the discectomy without implantation, while type II collagen significantly decreased. At the first month after implantation, type I collagen increase and type II collagen decreased. At 2 month after implantation, type I collagen decreased and type II collagen increased compared to the 2-month untreated groups. At 3 month after implantation, the staining level of both collagens returned to normal state and the ratio of type II to I collagen reached approximately 3.4 to 1, which is similar to the intact control.
